# Prevalence of *Listeria* Species on Food Contact Surfaces in Washington State Apple Packinghouses

**DOI:** 10.1128/AEM.02932-20

**Published:** 2021-04-13

**Authors:** Blanca Ruiz-Llacsahuanga, Alexis Hamilton, Robyn Zaches, Ines Hanrahan, Faith Critzer

**Affiliations:** a School of Food Science, Irrigated Agriculture Research and Extension Center, Washington State University, Prosser, Washington, USA; b Washington Tree Fruit Research Commission, Wenatchee, Washington, USA; The Pennsylvania State University

**Keywords:** *Listeria*, apples, environmental monitoring, food contact surface

## Abstract

The 2014 caramel apple listeriosis outbreak was traced back to cross-contamination between food contact surfaces (FCS) of equipment used for packing and fresh apples. For Washington state, the leading apple producer in the United States with 79% of its total production directed to the fresh market, managing the risk of apple contamination with Listeria monocytogenes within the packing environment is crucial. The objectives of this study were to determine the prevalence of *Listeria* spp. on FCS in Washington state apple packinghouses over two packing seasons and to identify those FCS types with the greatest likelihood to harbor *Listeria* spp. Five commercial apple packinghouses were visited quarterly over two consecutive year-long packing seasons. A range of 27 to 50 FCS were swabbed at each facility to detect *Listeria* spp. at two sample times, (i) postsanitation and (ii) in-process (3 h of packinghouse operation), following a modified protocol of the FDA’s *Bacteriological Analytical Manual* method. Among 2,988 samples tested, 4.6% (*n* = 136) were positive for *Listeria* spp. Wax coating was the unit operation from which *Listeria* spp. were most frequently isolated. The FCS that showed the greatest prevalence of *Listeria* spp. were polishing brushes, stainless steel dividers and brushes under fans/blowers, and dryer rollers. The prevalence of *Listeria* spp. on FCS increased throughout apple storage time. The results of this study will aid apple packers in controlling for contamination and harborage of L. monocytogenes and improving cleaning and practices for sanitation of the FCS on which *Listeria* spp. are the most prevalent.

**IMPORTANCE** Since 2014, fresh apples have been linked to outbreaks and recalls associated with postharvest cross-contamination with the foodborne pathogen L. monocytogenes. These situations drive both public health burden and economic loss and underscore the need for continued scrutiny of packinghouse management to eliminate potential *Listeria* niches. This research assesses the prevalence of *Listeria* spp. on FCS in apple packinghouses and identifies those FCS most likely to harbor *Listeria* spp. Such findings are essential for the apple-packing industry striving to further understand and exhaustively mitigate the risk of contamination with L. monocytogenes to prevent future listeriosis outbreaks and recalls.

## Abstract (precis)

Since 2014, fresh apples have been linked to outbreaks and recalls associated with postharvest cross-contamination with the foodborne pathogen L. monocytogenes. These situations drive both public health burden and economic loss and underscore the need for continued scrutiny of packinghouse management to eliminate potential *Listeria* spp. niches.

## INTRODUCTION

Washington state is the leading producer of apples in the United States (1), with 79% of its total apple production destined for fresh consumption (2). Because of the ubiquitous nature of the foodborne pathogen Listeria monocytogenes and its ability to grow under a wide array of environmental conditions (3), contamination of fresh apples can occur during preharvest, harvest, and postharvest (4), resulting in an increased risk of outbreaks of listeriosis. Cross-contamination during postharvest handling has been identified as the likely root cause of L. monocytogenes contamination in the 2011 cantaloupe and the 2014 caramel apple listeriosis outbreaks (5, 6), highlighting the need for robust environmental monitoring programs within packinghouses (7). In both outbreaks, L. monocytogenes contamination of food contact surfaces (FCS) associated with packing equipment was identified during environmental assessments (8–10).

In 2011, a listeriosis outbreak that was linked to cantaloupe resulted in 147 infected people and 33 deaths across 28 states (6). Of 39 environmental samples that were collected in the facility in Colorado, 13 tested positive for L. monocytogenes. Twelve of these 13 isolates matched outbreak-related strains and were taken from FCS, including brush and felt rollers (9).

In 2014, the multistate caramel apple listeriosis outbreak was the first outbreak related to whole fresh apples. This outbreak infected 35 people and left seven dead across 12 states (5). Of the 31 patients interviewed, 28 reported having eaten commercially prepackaged caramel apples, whereas three people reported having consumed only whole apples (8). In the FDA traceback investigation, six of the seven environmental samples positive for L. monocytogenes that matched outbreak strains were isolated from packing equipment FCS, such as brushes, conveyor belts, and a wooden bin; these results demonstrated the likely role cross-contamination of FCS played in subsequent apple contamination (8).

Since then, a listeriosis outbreak in 2017, with caramel apples as the presumptive source of contamination, infected three people (11). In addition, four voluntary recalls have been reported in the United States after random final product samples of whole apples (12, 13) and apple slices (14) or equipment surfaces (15) tested positive for L. monocytogenes. These outbreaks and recalls further emphasize the persistent risk of cross-contamination during the packing of fresh apples. Therefore, identifying strategies to control for contamination and harborage of L. monocytogenes is crucial.

As part of the Food Safety Modernization Act (FSMA), the FDA instated the implementation of a *Listeria* environmental monitoring program (EMP) as a preventive measure to further reduce the potential for foodborne outbreaks related to ready-to-eat (RTE) foods such as apples (16). Currently, the FDA does not recommend testing for a specific pathogen (e.g., L. monocytogenes) on FCS as part of an EMP. However, testing for indicator organisms on FCS is suggested. As indicator organisms, *Listeria* species can be used to identify the potential presence of L. monocytogenes by evaluating all six *Listeria* spp. of the *sensu stricto* group, including L. monocytogenes, Listeria ivanovii, Listeria innocua, Listeria seeligeri, Listeria welshimeri, and Listeria marthii (16). The detection of *Listeria* spp. on a surface does not necessarily indicate the presence of L. monocytogenes but rather that the conditions are suitable for the establishment and proliferation of L. monocytogenes (16).

Research evaluating the prevalence of *Listeria* spp. in different types of produce-packing facilities has been largely focused on both non-food contact surfaces (NFCS) (17–24) and the combined results of NFCS and FCS (25–31). However, research related to *Listeria* prevalence specifically on FCS is scarce; only two studies have been focused on FCS (32, 33), and neither was performed in apple packinghouses.

Thus, the goals of this research were (i) to determine the prevalence of *Listeria* spp. on FCS in Washington state apple packinghouses over two packing seasons and (ii) to identify in apple-packing facilities those FCS types and design features with the greatest likelihood to harbor *Listeria* spp.

## RESULTS AND DISCUSSION

### Prevalence of *Listeria* spp. in apple packinghouses.

The prevalence of *Listeria* spp. specifically on FCS was assessed over two packing seasons in Washington state apple packinghouses. *Listeria* spp. were isolated from all five packinghouses during both packing seasons. Among all tested samples (*n* = 2,988), 136 (4.6%) were confirmed positive for *Listeria* spp. (Table 1). To compare these results with those of previous studies that assessed the prevalence of *Listeria* spp. in different types of produce-processing facilities after 3 h of packinghouse operation (Table 2), the value of the in-process prevalence (7.2%) was used. Seven studies found similar results to our data even though different commodities and types of surfaces (NFCS versus FCS) were tested, with prevalence ranging from 5.5 to 10.8% (18, 19, 25, 29, 31–33). Only two studies specifically assessed FCS, which was the focus of this study, both finding similar rates of *Listeria* isolation in tomato (10.8%) (32) and frozen pepper (10.7%) (33) facilities.

**TABLE 1:** Prevalence of *Listeria* spp. by unit operation and sampling time

Unit operation	Examples of surfaces tested	No. of samples tested	Sampling time		Total prevalence (%)^*c*^
			Postsanitation^*a*^ (*n* = 1,497)	In-process^*b*^ (*n* = 1,491)	
Washing (dump tank/flume)	Dump tank, flumes, PVC rollers, traction belting	285	0 A^*d*^	1.4 A	0.7 A
Washing/sanitizing/rinsing (spray bars)	Brush rollers, plastic flaps, side edges	331	0.6 A	1.8 A	1.2 A
First drying (fan and/or blower)	Brush rollers, dividers	394	4.6 B	14.2 CD	9.4 C
Wax coating	Polishing brushes, plastic flaps, transfer points	110	10.9 B	23.6 D	17.3 D
Second drying (tunnel dryer)	Dryer rollers, bristle rollers, transfer points	304	4.6 B	11.8 BC	8.2 C
Sorting	Sorter cups, interlocking conveyor belts, solid conveyor belts, plastic guide rails, side edges, Teflon tapes, transfer points	1,254	0.8 A	6.9 B	3.8 B
Packing	Packing tables, solid conveyor belts, plastic crates, plastic flaps	310	0 A	0.7 A	0.3 A
Total	2,988	1.9	7.2	4.6

a Statistical analysis of postsanitation prevalence. Fisher’s exact test, *P* < 0.001.

b Statistical analysis of in-process (3 h of production) prevalence. Fisher’s exact test, *P* < 0.001.

c Statistical analysis of total prevalence. Chi-square test, *P* < 0.001.

d Values within a column that are not followed by the same letter are significantly different (*P* < 0.001).

**TABLE 2:** Prevalence of *Listeria* spp. and L. monocytogenes on food contact surfaces and non-food contact surfaces in different types of produce packinghouses or processing facilities

Type of produce	Prevalence (%)		Type of surface tested	Reference
	*Listeria* spp.	Only L. monocytogenes		
Tree fruits	N/A^*a*^	56.4	NFCS	17
Cabbage, beets, parsnips	6.8	4.0	NFCS	18
Microgreens, peach, apple, tomato, broccoli, cauliflower, cucumber	5.8	3.2	NFCS	19
Packinghouses and fresh-cut facilities	3.4	3.0	NFCS	20
Fresh-cut vegetables	N/A	7.9	NFCS	21
Vegetables	N/A	9.5	NFCS	22
Mushrooms	25.1	18.8	NFCS	23
Potatoes	50.7	3.0	NFCS	24
Avocadoes	8.7	N/A	Both	25
Fresh-cut vegetables	N/A	4.4	Both	26
Mushrooms	15.7	1.6	Both	27
Frozen vegetables (e.g., cauliflower, mushrooms, broccoli, carrot, zucchini)	82.2	41.3	Both	28
Frozen vegetables (e.g., tomato, broccoli, carrot, spinach, artichoke)	7.8	1.2	Both	29
Prepackaged salad, canned vegetables	N/A	5.4	Both	30
Cabbage	5.5	2.1	Both^*b*^	31
Tomatoes	10.8	N/A	FCS	32
Frozen peppers	10.7	0	FCS	33

a N/A, information not available.

b Results of *Listeria* prevalence are not combined for both types of surfaces. Values account for FCS only.

The reported prevalence of *Listeria* spp. was highest in packinghouses that processed frozen vegetables (82.2%) (28), potatoes (50.7%) (24), and mushrooms (23.9% [23] and 15.7% [27]). Unlike tree fruit, such commodities grow directly in contact with soil, which may increase the likelihood of finding *Listeria.* Other studies have reported a greater prevalence of *Listeria* spp. probably due to having evaluated either both NFCS and FCS or only NFCS, such as floors and drains, where *Listeria* isolation is more likely.

While this study did not specifically target L. monocytogenes given that FCS were tested, in studies that did, rates of isolation generally fell between 1.2 and 9.5%. Higher rates of L. monocytogenes were reported in three studies, targeting NFCS primarily in fresh-cut mushrooms (18.8%) (23), frozen vegetables (41.3%) (28), and tree fruits (56.4%) (17). Differences in frequency and stringency of sanitation programs, implementation of environmental monitoring programs targeting *Listeria*, and growing region may be significant divers of *Listeria* isolation rather than commodity type. Other factors, such as experimental design, sampling methods, and the size and age of packinghouses tested may have also impacted outcomes (19).

### The prevalence of *Listeria* spp. was affected by unit operation and FCS type.

The prevalence of *Listeria* spp. in each unit operation is displayed in Table 1. *Listeria* spp. were most frequently isolated from the wax coating unit operation (17.3%; *n* = 110) (*P* < 0.001). Furthermore, throughout the apple-packing process, the four FCS that showed the greatest prevalence of *Listeria* spp. were polishing brushes (19.6%; *n* = 92), dividers under fans/blowers (17.4%; *n* = 46), dryer rollers (10.5%; *n* = 143), and brushes under fans/blowers (9.7%; *n* = 206) (*P* < 0.001) (Table 3).

**TABLE 3:** Frequency of isolation of *Listeria* spp. by food contact surface

Food contact surface(s)	No. of samples tested	Frequency (%)
Polishing brushes (e.g., polyethylene, polypropylene, nylon, horsehair mix)	92	19.6 A^*a*^
Stainless steel dividers under fan/blowers^*b*^	46	17.4 AB
Dryer rollers (e.g., stainless steel roller wrapped with vinyl or Teflon)	143	10.5 ABC
Brushes under fan/blower (e.g., polyethylene, polypropylene)	206	9.7 ABC
Bristle rollers (e.g., polyethylene, polypropylene)	160	8.8 BCD
Plastic interlocking chain conveyor belts (e.g., polypropylene, polyethylene)	256	5.1 CDE
Teflon transfer points and tape^*b*^	304	4.6 CDE
Plastic flaps and transfer points (e.g., PVC, polyurethane)^*b*^	427	4.2 DE
Side edges (e.g., painted-steel or high-density polyethylene)	123	3.3 CDEF
Sorter cups^*b*^	76	2.6 CDEF
Solid conveyor belts (e.g., PVC, polyurethane, polyester nylon)	186	1.6 EF
Sorting plastic guide rails	128	1.6 EF
Traction belting (e.g., polyurethane, polyester nylon)^*b*^	66	1.5 CDEF
Brushes under spray bars (e.g., polyethylene, polypropylene)	227	0.9 F
Stainless steel dump tank and flume	108	0.9 EF
PVC rollers	123	0.8 EF
Packing tables and plastic crates	64	0.0 EF
Cup droppers (e.g., painted steel)^*b*^	60	0.0 EF
Sorting brushes (e.g., polyethylene, polypropylene)	193	0.0 F

a Values within a column that are not followed by the same letter are significantly different (*P* < 0.001).

b Food contact surfaces that had a surface area smaller than 0.93 m^2^.

In the wax coating unit operation, polishing brushes were the FCS most commonly implicated. These findings suggested a deficiency of routine sanitation procedures at this sampling site (16) and the ability of these FCS to trap wax residues and *Listeria* cells within polishing brush bristles. In the 2014 caramel apple listeriosis outbreak, polishing brushes were one of the FCS that L. monocytogenes was isolated from (8). Likewise, similar findings were discussed in an annual fruit and vegetable convention (34), where a higher prevalence of *Listeria* spp. was reported in the wax coating area, indicating that wax residues are related to an increase in persistence of *Listeria* spp. on both FCS and NFCS.

Studies that support our results have reported a greater long-term survival of L. monocytogenes on waxed apples than on unwaxed apples due to moisture retention over time (34), ultimately suggesting that entrapment of L. monocytogenes cells and moisture within a wax coating were conducive for forming a microenvironment that enhances the survival of *Listeria* in apples (35) and E. coli O157:H7 cells that were found embedded in wax platelets on apples (36). Another factor that could explain our results is the pH level of commercial waxes (6.7 to 8.6) (37). The optimal pH level for *Listeria* to grow is 7.0 (38); therefore, if sufficient water activity, nutrients, and temperature are maintained, wax residues on FCS and NFCS may support the growth of *Listeria* spp. if not otherwise removed.

Conversely, other studies have reported the immediate antibacterial activity of wax application on apples against L. monocytogenes (34), E. coli O157:H7 (37), and Salmonella enterica serovar Muenchen (37), possibly because of one of the components of commercial wax, either isopropyl or ethyl alcohol (34). However, the concentration of these components in wax ranges from 15% to 23% (39), which otherwise volatilizes rapidly and does not have an antimicrobial effect over time. In an *in vitro* study, commercial wax did not show bactericidal activity against L. monocytogenes (40).

In contrast, a study performed on NFCS in tree fruit packinghouses reported that the incidence of L. monocytogenes under brush beds and in first drying and wax coating areas was not significantly different (17). These differences may be a result of NFCS being very interconnected between these unit operations, many times sharing drains.

Ultimately, future studies are warranted to further elucidate the mechanisms best suited for cleaning polishing brushes and determining if there are conditions where *Listeria* growth can be supported in this unit operation. Whether driven by wax accumulation during packing or *Listeria* growth events, the waxing unit operation is one that should be more closely scrutinized in order to limit cross-contamination of apples during packing.

The second highest prevalence of *Listeria* spp. was obtained from both the first drying operation (9.4%; *n* = 394) and the second drying (tunnel dryer) (8.2%; *n* = 304) unit operations (*P* < 0.001). In the first drying unit operation, dividers and brush rollers located underneath fans/air blowers (NFCS) were the FCS that showed the greatest prevalence of *Listeria* spp. Migration of pathogens from zones 2 or 3 (NFCS) to zone 1 (FCS) has been reported in previous studies (41, 42). As fans and air blowers circulate air, they also spread pathogens contained on the blades, motor, and cover of the fan, leading to cross-contamination of the dividers and brush rollers. Moreover, repeated isolation of *Listeria* spp. has been shown on fans over brush beds in produce packinghouses (20) and on freezer fans in meat facilities (43). These devices represent potential niches for L. monocytogenes (44) and are recommended to be scheduled into daily cleaning and sanitation programs (7).

In the second drying unit operation, dryer rollers were the FCS that were most implicated. Tunnel dryer operating temperatures of 30 to 50°C may create opportunities for *Listeria* growth in niches if other growth conditions are met. The optimal growth temperature of L. monocytogenes is 30 to 37°C (45), and it can also grow at temperatures up to 50°C (3). Packinghouses in this study often operated within the range of the optimal growth temperatures, thus increasing the potential proliferation of *Listeria* over time. Correspondingly, the survival of *L. innocua* (46), E. coli O157:H7 (37), and *Salmonella* Muenchen (37) has been reported on apples that were exposed to similar drying conditions.

Other explanations included visible dried leaf buildup in the inlet and outlet of the tunnel dryer. Also, in some packinghouses, a brush roller that was in the interior of the dryer to clean out leaves from the dryer rollers was considered a point of cross-contamination. Stainless steel dryer rollers were wrapped with different materials including vinyl or Teflon. Cracks and worn edges were observed in most of these FCS. One packinghouse used nonwrapped rollers, and *Listeria* spp. were never isolated from this surface. Further research is warranted to determine growth/no-growth conditions within the tunnel drying unit operation and if surface type for rollers plays a significant role.

The third highest prevalence of *Listeria* spp. was obtained from the sorting unit operation (3.8%; *n* = 1,254). Bristle rollers (8.8%; *n* = 160), plastic interlocking chain conveyor belts (5.1%; *n* = 256), Teflon transfer points and tape (4.6%; *n* = 304), plastic flaps and transfer points (4.2%; *n* = 427), side edges (3.3%; *n* = 123), sorter cups (2.6%; *n* = 76), solid conveyor belts (1.6%; *n* = 186), and plastic guide rails (1.6%; *n* = 128) were the FCS that were most implicated.

Interlocking belts are hard to clean due to their continuous length and joints in belt links causing entrapment of bacteria more easily (47, 48). Similarly, the rough polymeric material and hygienic design of solid conveyor belts support growth and bacterial adhesion (49). Studies performed in a blueberry-packing line (48), a minimally processed vegetable plant (49), and a sandwich processing plant (50) have reported that solid conveyor belts in their sorting areas were the major source of microbial contamination. Also, different species of *Listeria* were isolated from conveyor belts including *L. ivanovii* in frozen pepper packinghouses (33) and L. monocytogenes, Listeria grayi, and *L. innocua* in a cabbage-packing facility (31). In 2013, the FDA evaluated the prevalence of *Listeria* spp. in 17 cantaloupe-packing facilities, and two samples collected from conveyors were positive for L. monocytogenes at one facility (51).

Plastic flaps used to slow fruit down and transfer points used to bridge movement between one conveyor surface to the next are commonly made of polyvinylchloride (PVC), polyurethane, or Teflon, which have a hydrophobic nature allowing for easier microbial attachment (48, 52).

**FIG 1:**
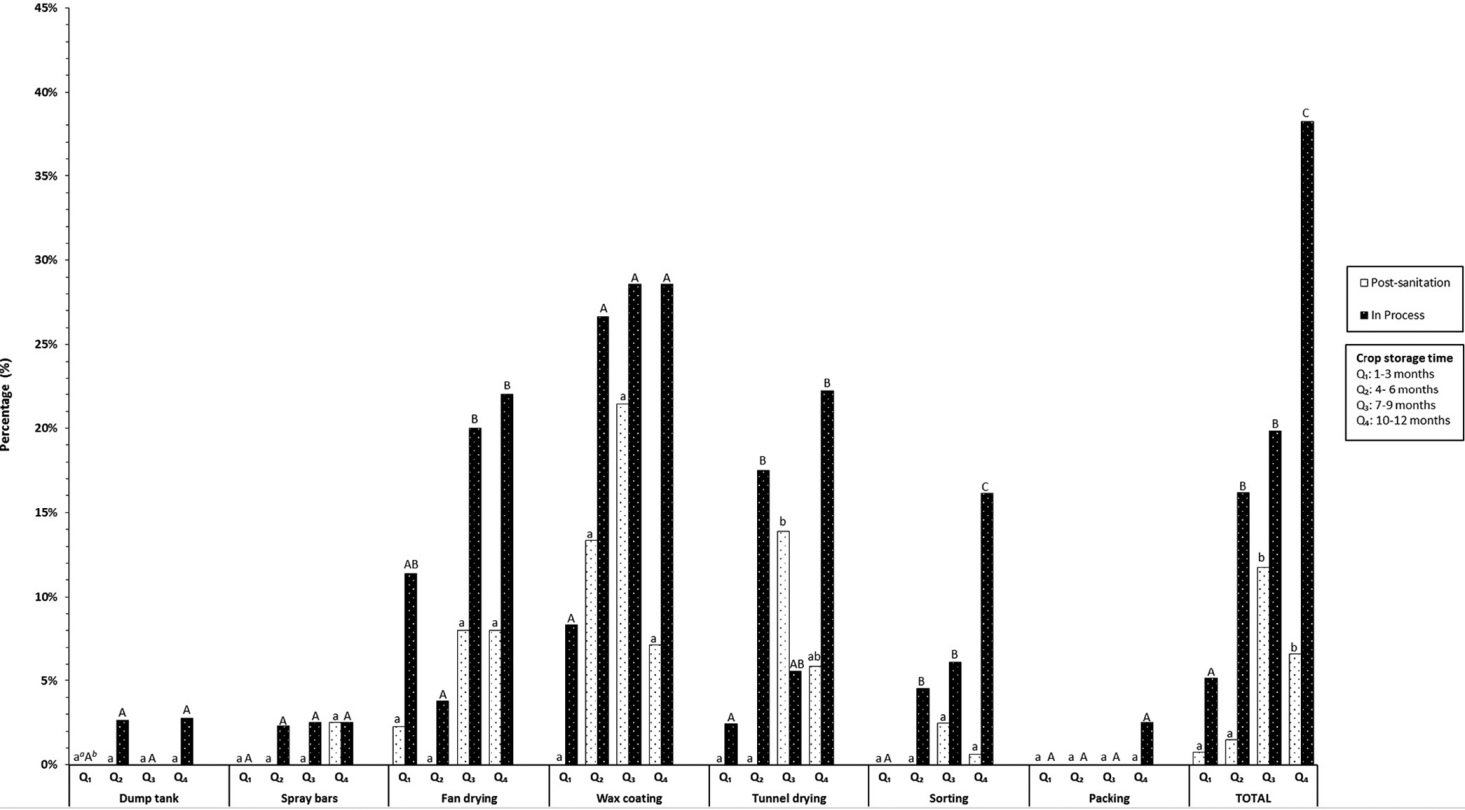
Prevalence of *Listeria* spp. is affected by unit operation and crop storage time. Footnotes: *^a^*, postsanitation bars within each unit operation that are not followed by the same lowercase letter are significantly different (*P* < 0.05); *^b^*, in-process bars within each unit operation that are not followed by the same uppercase letter are significantly different (*P* < 0.05).

In addition, at the sorting unit operation, a significant increase in the prevalence of *Listeria* spp. was observed in the last sampling period (Q_4_) during the in-process sampling (16.1%; *n* = 161; *P* < 0.05) (Fig. 1). This result could be mainly attributed to cross-contamination with incoming apples that were stored for a longer period (10 to 12 months), which results in high rates of apple decay and potentially higher populations of *Listeria* coupled with the fact that wax may still be solidifying up until this unit operation.

Lastly, the lowest prevalence of *Listeria* spp. was obtained from the washing (0.7%; *n* = 285), washing/sanitizing/rinsing (1.2%; *n* = 331), and packing (0.3%; *n* = 310) (*P* < 0.001) unit operations. Sites that were exposed to sanitizers throughout production (brushes under spray bars [0.9%; *n* = 227], dump tank/flume [0.9%; *n* = 108]), as well as side edges (3.3%; *n* = 123), sorter cups (2.6%; *n* = 76), solid conveyor belts (1.6%; *n* = 186), sorting guide rails (1.6%; *n* = 128), traction belting (1.5%; *n* = 66), PVC rollers (0.8%; *n* = 123), packing tables and plastic crates (0.0%; *n* = 64), sorting brushes (0.0%; *n* = 193), and cup droppers (0.0%; *n* = 60) had the lowest occurrence of *Listeria* spp.

The low prevalence of *Listeria* spp. obtained in the dump tank and flume (washing unit operation) was attributed to the use of sanitizers (chlorine or peracetic acid [PAA]) within the water in the dump tank and connected flume. These sanitizers ensure the adequate microbial quality of wash water and reduce the likelihood of cross-contamination by inactivating foodborne pathogens that are introduced into the water (53, 54). The efficacy of chlorine (55–57) and PAA (58, 59) to avoid cross-contamination with L. monocytogenes has already been demonstrated in produce wash water. Although, during packing operations, incoming organic matter such as soil, leaves, and decaying fruit can reduce the efficacy of the sanitizer over time (60, 61). In the 2011 cantaloupe listeriosis outbreak, the use of municipal water without sanitizer in the wash water was one of the factors that was identified as a likely cause of cross-contamination of L. monocytogenes to cantaloupe (9).

Packinghouses that participated in this study used test strips or titration kits to monitor the concentration of these sanitizers within the dump tank and flume systems in addition to continuous monitoring and dosing systems. Dump tanks and flumes were all made of stainless steel. In equipment made of this type of material, L. monocytogenes cells have shown the least resistance to sanitizers, including PAA, chlorine dioxide, and acidic quaternary ammonia (62).

Isolation of *Listeria* spp. continued to be relatively low once product was conveyed out of the flume to the brush bed (washing/sanitizing/rinsing unit operation) where soap and sanitizers (e.g., PAA, chlorine, ozone, and chlorine dioxide) were applied via spray bars. The incorporation of sanitizer at this unit operation, similar to that of the dump tank and flume, safeguards against cross-contamination between apples and brush rollers; thus, low recovery should be anticipated. The use of PAA has been shown to reduce cross-contamination between apples inoculated with a surrogate of L. monocytogenes and brush beds of commercial apple packinghouses (63). Chlorine (64), chlorine dioxide (61), and ozone (61) have been demonstrated to reduce L. monocytogenes counts on apples.

Brush rollers have been identified as common harborage sites for *Listeria* because of their complex hygienic design (44, 65). These FCS can entrap apple debris and retain moisture within their bristles, creating the right environment for *Listeria* to grow. In the 2011 listeriosis cantaloupe outbreak, brush rollers were one of the FCS where L. monocytogenes was isolated (9). However, no specification of the location of the brush beds was provided. Brush rollers that were evaluated within this unit operation were located in between spray bars that dispensed sanitizers and soap and were composed of polypropylene or polyethylene with a staple set cylinder configuration. Only two sites (1%; *n* = 197) were positive for *Listeria* spp. at this unit operation during the in-process sampling, demonstrating that any shortcomings of brush sanitary design can be controlled for through regular introduction of sanitizers during production.

The low prevalence of *Listeria* spp. reported at the packing unit operation could be explained by less carryover of *Listeria*. As demonstrated in the previous unit operations, wax residues and *Listeria* microbial load of incoming apples were able to cross-contaminate FCS until the sorting unit operation, with wax set by the time apples are transferred to packing lines, potentially allowing for less transfer from apples to packing surfaces.

### Prevalence of *Listeria* spp. was affected by the timing of sampling (postsanitation, in-process).

Of the 1,497 postsanitation samples, 1.9% were positive for *Listeria* spp. compared to 7.2% of the 1,491 in-process samples (Table 1). Among all of the positive *Listeria* species samples, 21% (*n* = 28) were detected during the postsanitation sampling, whereas 79% (*n* = 108) were detected during the in-process sampling.

In addition, timing of *Listeria* species isolation was also evaluated for each site among all of the positive samples that were positive during a sampling event based on three scenarios, (i) the sampling site testing positive postsanitation and negative in-process, (ii) the sampling site testing negative postsanitation and positive in-process, or (iii) the sampling site testing positive both during postsanitation and in-process, to determine the frequency of each (Table 4). The outcomes of each scenario were significantly different from each other (*P* < 0.001), with *Listeria* species-positive sites most frequently positive only for the in-process sampling (scenario 2) (75.9%). This could be explained by allowing *Listeria* to come out of niche sites and subsequently contaminate FCS during the 3 h of packinghouse operation (16). Another rationale is that incoming crop got to the packing line with a *Listeria* load capable of cross-contaminating the FCS. Similarly, in a study performed in an avocado packinghouse, which assessed the prevalence of *Listeria* spp. over two sampling times (25) as performed in this research, a higher prevalence of *Listeria* spp. during the in-process sampling was attributed to cross-contamination with the avocadoes during processing (25). More attention should be paid to this question in order to identify the most opportune approach, which can be applied to detect harborage within packing equipment by running the equipment without any crop.

**TABLE 4:** Frequency of *Listeria* spp. isolation for a specific sampling site based on sampling time during a sampling event

Scenario	Result at sampling time		No. of sampling sites	Frequency (%) (*n* = 116)^*a*^
	Postsanitation	In-process		
1^*b*^	Positive	Negative	8	6.9 A^*e*^
2^*c*^	Negative	Positive	88	75.9 C
3^*d*^	Positive	Positive	20	17.2 B

a Total number of sampling sites with at least one positive detection of *Listeria* spp. From the total of positive samples (*n* = 136), 20 sampling sites belonged to scenario 3, thus total *n* = 116.

b Sampling sites in which *Listeria* spp. were detected only in the postsanitation sampling.

c Sampling sites in which *Listeria* spp. were detected only in the in-process sampling.

d Sampling sites in which *Listeria* spp. were detected in both postsanitation and in-process samplings.

e Values within a column that are not followed by the same letter are significantly different (*P* < 0.001).

Isolation from surfaces in both sampling events (scenario 3) occurred 17.2% of the time and is evidence of a deficiency of cleaning and sanitation procedures. This scenario was mostly reported on surfaces such as brushes (45%), including polishing brushes, bristle rollers, and brush rollers under the fan. Secondly, 15% of the cases were reported in dryer rollers, with many showing increased wear at this unit operation, resulting in cracks.

The low prevalence of *Listeria* spp. found in scenario 1 (6.9%) was attributed to a sample collection of different sampling sites (such as rollers inside the tunnel dryer). Other explanations include removal during prior sampling or during packing or application of sanitizers (such as from spray bars and fruit) that inactivated initial contamination.

### The prevalence of *Listeria* spp. increased throughout crop storage time.

Overall, an increase in the prevalence of *Listeria* spp. on FCS was observed throughout crop storage time and during both sampling times (Fig. 1). The highest prevalence of *Listeria* spp. was obtained during the last quarter of sampling (Q_4_) in the in-process sampling (38.2%; *P* ≤ 0.05). After Q_2_, a significantly higher prevalence of *Listeria* spp. was observed at both sampling times.

However, the increasing prevalence of *Listeria* spp. throughout crop storage time (quarters) differed by unit operation. For the wax coating unit operation, a greater prevalence of *Listeria* spp. was obtained during all four quarters at both sampling times, and it did not significantly increase over time. The only unit operation where the prevalence of *Listeria* spp. increased during the postsanitation sampling was tunnel drying (from Q_1_ = 0% to Q_3_ = 13.9%; *P* ≤ 0.05), and the three unit operations that accounted for the increase of the in-process prevalence of *Listeria* spp. over storage time were fan drying, tunnel drying, and sorting. These unit operations showed significantly higher frequencies of isolation after the first quarter of sampling.

According to our findings, the prevalence of *Listeria* spp. increased after 6 months and 3 months of storage time for the postsanitation and in-process sampling, respectively. One factor that could have influenced the increase during the postsanitation sampling is the type of *Listeria* species that persisted in the packing equipment (66), affecting the efficacy of the cleaning and sanitation procedures. Conversely, the increase in the prevalence of *Listeria* spp. during the in-process sampling was principally attributed to cross-contamination between apples and FCS. Throughout storage, some of the most common apple postharvest decay diseases caused by the fungi Botrytis cinerea, Penicillium expansum, and Mucor piriformis (67) can increase microbial pathogen growth (68). After harvest, apple bins go through a fungicide drenching step before being stored for up to 12 months (54). Nevertheless, drenching methods can cause cross-contamination with pathogens including L. monocytogenes due to the reuse of fungicide solution (54). In addition, there is not a culling step (to eliminate bruised or damaged apples) before the storage. Punctures, wounds, or damaged skin caused during harvest and transportation facilitate the spread and growth of bacteria and fungus (69). Fungal growth surrounding bruised tissues degrade the protective epidermal layer (70) and produce a pH gradient (due to the use of organic acids) neutralizing the apple flesh (4) and leading to the potential for survival and growth of *Listeria*. Thus, it has been hypothesized that as the storage time increases so does the fungal growth and internal fruit pH, and when combined, these two factors lead to an increase of the *Listeria* microbial load. However, further investigation regarding the relationship between the survival of *Listeria* and fungal postharvest disease is required in a longer-term storage setting.

Moreover, *Listeria* can grow under refrigerated temperatures (71) employed for both regular atmosphere (RA) and long-term controlled atmosphere (CA) storage of apples. L. monocytogenes uses different cold adaptation mechanisms, such as the stress response gene sigma factor B (*sigB*), induced by refrigerated temperatures (72). This gene promotes the formation of cryoprotectants (i.e., glycine betaine and carnitine), which stimulate cell proliferation under cold stress (72, 73). Another mechanism is the alteration of the cellular membrane lipid composition, in which the amount of unsaturated fatty acids increases under refrigerated temperatures to ensure the optimum membrane fluidity, enzyme activity, and transportation of solutes necessary for *Listeria* survival (73, 74).

Studies that evaluated the survival of *Listeria* on apples throughout different long-term storage scenarios reported the survival of L. monocytogenes on apples after 3 months (75) and 5 months (34) of RA storage. Also, after 7 months of either RA or CA storage, *L. innocua* survived on Fuji apples (76). It has been reported that CA storage reduces aerobic bacterial growth due to a reduced availability of oxygen (77), though facultative anaerobic bacteria such as *Listeria* cannot be inhibited under these conditions. Seven months of CA storage resulted in a greater reduction of *L. innocua* populations than RA storage (76). However, CA treatment did not significantly influence populations of L. monocytogenes (68) and *L. innocua* (76).

These findings provide science-based information on the FCS that require the most attention in order to not become a source of *Listeria* species contamination in apple packinghouses. Such results will provide a better understanding of how to control for contamination of L. monocytogenes to prevent future foodborne outbreaks and recalls associated with fresh apples through the improvement of EMPs, as well as enhanced cleaning and sanitation procedures on the most *Listeria*-prevalent FCS. Lastly, several areas of future research have been identified in order to determine the ability of *Listeria* to survive and grow in wax and the complex nature of *Listeria* survival and growth on apples throughout storage, considering the interconnectedness to decay-causing organisms.

## MATERIALS AND METHODS

### Apple packinghouse selection and layout.

Five commercial apple-packing facilities located in eastern Washington state, United States, with varying line design and cleaning practices were chosen for this study. The distance range between apple packinghouses was from approximately 14 to 190 km. The selection of packinghouses was based on the packers’ willingness to participate in learning about their operation without added cost. The identity of packinghouses was kept confidential.

The product flow within each packinghouse was diagramed. Wet and dry areas were identified based on the presence of water during operations. The apple-packing process was divided into seven unit operations as follows: washing, washing/sanitizing/rinsing, fan drying, wax coating, tunnel drying, sorting, and packing (78) (see Fig. S1 in the supplemental material).

### Sample sites.

Food contact surfaces (zone 1) on different apple packinghouse equipment were selected from each unit operation (see Table 1; see also Table S1 in the supplemental material). In accordance with the recommendations of the FDA (16), sampling sites were chosen based on environmental conditions and operations that support the growth of *Listeria* (i.e., hygienic design features, material type, efficacy and frequency of cleaning and sanitation procedures, and packer needs). Between 27 and 50 sites were sampled at each facility. Exact sampling sites for each packinghouse were photographed and described in detail to ensure consistency during all sampling events.

### Sample collection.

Each packinghouse was visited four times over each of two packing seasons for a total of eight data collection points per facility. The purpose of these visits was to obtain data about FCS throughout the year-long packing season. Generally, apples are stored in cold storage rooms for up to 12 months before packing. Packing season 1 (apple crop 2018) included apples harvested from September through November 2018 and stored until July 2019, whereas packing season 2 (apple crop 2019) included apples harvested from September through November 2019 and stored until July 2020.

Sampling periods were divided into four quarters. For instance, quarter 1 (Q_1_) represented apples stored for 1 to 3 months, quarter 2 (Q_2_) represented apples stored for 4 to 6 months, quarter 3 (Q_3_) represented apples stored for 7 to 9 months, and quarter 4 (Q_4_) represented apples stored for 10 to 12 months. Typically, commercial apple-packing facilities in Washington state use refrigerated regular atmosphere (RA, 0 to 2°C) for apples that are stored for up to 3 months, while controlled atmosphere (CA, 0 to 2°C; O_2_, 1 to 4%; CO_2_, 0 to 2%) is used for apples stored for up to 12 months. 1-Methylcyclopropene (1-MCP) is employed on all varieties of apples, regardless of storage treatment, except organically produced fruit.

Sample collection occurred at two sampling times as follows: (i) after cleaning and sanitation procedures (postsanitation) and (ii) after 3 h of packinghouse operation (in-process). A 0.93-m^2^ (30.5 cm by 30.5 cm) surface area was sampled with a premoistened sponge sampling stick (EZ-Reach sponge samplers with 10 ml of Dey-Engley [D/E] neutralizing broth; World Bioproducts LLC, Woodinville, WA). Sampling sites with smaller surface areas were swabbed entirely. Samples were collected against standard product flow from dry areas to wet areas to avoid cross-contamination.

All collected samples were transported in a refrigerated cooler and analyzed within 24 h of collection in the Food Microbiology Laboratory of the Irrigated Agriculture Research and Extension Center at Washington State University, Prosser, WA.

### Isolation, detection, and confirmation of *Listeria* spp.

The isolation, detection, and confirmation of *Listeria* spp. were conducted following a modified FDA *Bacteriological Analytical Manual* (BAM) method (79). Each sampling sponge was hand-massaged and enriched with 90 ml of buffered *Listeria* enrichment broth (BLEB; Difco, Becton, Dickinson Co., Sparks, MD) for 4 h at 30°C. In order to select for *Listeria* spp., 1 ml of each of the following antibiotics, previously rehydrated and filter-sterilized, was added to the broth as follows: 10 mg/liter acriflavin monohydrochloride (Acros Organics, Fair Lawn, NJ), 40 mg/liter nalidixic acid (Alfa Aesar, Ward Hill, MA), and 50 mg/liter cycloheximide (Acros Organics, Fair Lawn, NJ). Samples were enriched for an additional 44 h at 30°C. Ten microliters of enrichment was streaked in duplicate onto modified Oxford medium (MOX; Difco, Becton, Dickinson Co., Sparks, MD) containing the modified Oxford antimicrobic supplement (Bacto; Becton, Dickinson Co., Sparks, MD) and incubated for 48 h at 35°C. Based on characteristic esculin hydrolysis (black halo formation), presumptive *Listeria* colonies were selected for DNA extraction and PCR confirmation. Selected colonies were suspended in 0.5 ml of Tris-EDTA 1× buffer solution (Fisher Scientific, Fair Lawn, NJ) and stored at 4°C until the extraction of DNA. DNA extraction from presumptive *Listeria* colonies was conducted using a GenElute bacterial genomic DNA kit (Sigma-Aldrich, St. Louis, MO). A PCR amplification of a 1,300-bp target region in the *iap* gene was performed in the DNA extracted from presumptive positive *Listeria* species colonies. A pair of primers for the isolation of L. monocytogenes, *L. ivanovii*, *L. innocua*, *L. seeligeri*, and *L. welshimeri* was utilized (forward sequence, 5′-ATATGAAAAAAGCAACTATCGC-3′, and reverse sequence, 5′-AGAATACTAAATCACCAGGTTTTGC-3′; Thermo Fisher Scientific, Foster City, CA) (78). PCR assay was conducted using DreamTaq green PCR master mix (2×) (Thermo Fisher Scientific, Foster City, CA) in a 50-μl reaction mixture. Each 50-μl reaction mixture contained 25 μl of DreamTaq green PCR master mix, 1 μl of forward primer (10 μM), 1 μl of reverse primer (10 μM),18 μl of molecular-grade water, and 5 μl of template DNA. An isolate of Listeria innocua 33090 (American Type Culture Collection, Manassas, VA) and molecular-grade water (Sigma-Aldrich, St. Louis, MO) were used as positive and negative controls, respectively. All components were added to low-profile 8-tube strips (0.2 ml) with individually attached caps (Greiner Bio-One, Germany). Thermocycling was performed in the Mastercycler Nexus (Eppendorf, Germany). A 35-cycle program was run at 95°C for 30 s (denaturation), 62°C for 30 s (annealing), and 72°C for 1 min (elongation), followed by a 4°C hold until amplified products were evaluated. PCR products were analyzed by gel electrophoresis and visualized in E-Gel EX 1.0% agarose gel (Thermo Fisher Scientific, Foster City, CA). Each cell of the gel contained 5 μl of amplified DNA and 15 μl of molecular-grade water (Sigma-Aldrich, St. Louis, MO). A 1-kb DNA-molecular ladder (Thermo Fisher Scientific, Foster City, CA) was included for comparison of amplicon size. Electrophoresis was carried out for 10 min at 48 V and 90 W. A positive result for *Listeria* spp. was indicated by the presence of characteristic bands at 1,300 bp.

This approach was used to identify only *Listeria sensu stricto* as a group (*Listeria* species including L. monocytogenes, *L. ivanovii*, *L. innocua*, *L. seeligeri*, and *L. welshimeri*). Further evaluation of isolates was not conducted, as agreed upon by participants in the survey.

### Statistical analysis.

A chi-square test or Fisher’s exact test (when expected observations were lower than 5) was used to analyze the categorical data of the presence or absence of *Listeria* spp. based upon the following categorical variables: unit operations (washing, washing/sanitizing/rinsing, fan drying, wax coating, tunnel drying, sorting, and packing), timing of sampling (postsanitation and in-process), sampling periods (Q_1_, Q_2_, Q_3_, and Q_4_), and type of FCS (e.g., brushes under fans, polishing brushes, dryer rollers, bristle rollers, dump tank, and plastic flaps). A *post hoc* pairwise comparison was used to compare the levels of each categorical variable when a significant difference was observed. The significance level for all tests was α = 0.05. Statistical analysis was performed in R (version 4.0.2) using RStudio (version 1.3.1056) (RStudio, Inc., Boston, MA, USA).

## Supplementary Material

Supplemental file 1

## References

[1] U.S. Department of Agriculture, National Agricultural Statistics Service, Northwest Regional Field Office. 2019. 2019 Washington annual statistical bulletin. U.S. Department of Agriculture, Washington, DC.

[2] U.S. Department of Agriculture, National Agricultural Statistics Service. 2019. 2019 State agriculture overview Washington. U.S. Department of Agriculture, Washington, DC.

[3] Farber JM, Peterkin I. 1991. *Listeria monocytogenes*, a food-borne pathogen. Microbiol Rev 55:476–511. doi:10.1128/MR.55.3.476-511.1991. 1943998 PMC372831

[4] Beuchat LR. 2002. Ecological factors influencing survival and growth of human pathogens on raw fruits and vegetables. Microbes Infect 4:413–423. doi:10.1016/s1286-4579(02)01555-1. 11932192

[5] Centers for Disease Control and Prevention. 2015. Multistate outbreak of listeriosis linked to commercially produced, prepackaged caramel apples made from Bidart Bros apples (final update). Centers for Disease Control and Prevention, Atlanta, GA.

[6] Centers for Disease Control and Prevention. 2012. Multistate outbreak of listeriosis linked to whole cantaloupes from Jensen farms, Colorado (final update). Centers for Disease Control and Prevention, Atlanta, GA.

[7] United Fresh Produce Association. 2018. Guidance on environmental monitoring and control of Listeria for the fresh produce industry. United Fresh Produce Association, Washington, DC.

[8] Angelo KM, Conrad AR, Saupe A, Dragoo H, West N, Sorenson A, Barnes A, Doyle M, Beal J, Jackson KA, Stroika S, Tarr C, Kucerova Z, Lance S, Gould LH, Wise M, Jackson BR. 2017. Multistate outbreak of *Listeria monocytogenes* infections linked to whole apples used in commercially produced, prepackaged caramel apples: United States, 2014–2015. Epidemiol Infect 145:848–856. doi:10.1017/S0950268816003083. 28065170 PMC6542465

[9] McCollum JT, Cronquist AB, Silk BJ, Jackson KA, O'Connor KA, Cosgrove S, Gossack JP, Parachini SS, Jain NS, Ettestad P, Ibraheem M, Cantu V, Joshi M, DuVernoy T, Fogg NW, Gorny JR, Mogen KM, Spires C, Teitell P, Joseph LA, Tarr CL, Imanishi M, Neil KP, Tauxe RV, Mahon BE. 2013. Multistate outbreak of listeriosis associated with cantaloupe. N Engl J Med 369:944–953. doi:10.1056/NEJMoa1215837. 24004121

[10] U.S. Food and Drug Administration. 2011. Environmental assessment: factors potentially contributing to the contamination of fresh whole cantaloupe implicated in a multi-state outbreak of listeriosis. U.S. Food and Drug Administration, Washington, DC.

[11] Marus JR, Bidol S, Altman SM, Oni O, Parker-Strobe N, Otto M, Pereira E, Buchholz A, Huffman J, Conrad AR, Wise ME. 2019. Notes from the field: outbreak of listeriosis likely associated with prepackaged caramel apples—United States, 2017. MMWR Morb Mortal Wkly Rep 68:76–77. doi:10.15585/mmwr.mm6803a5. 30677010

[12] U.S. Food and Drug Administration. 2017. Jack Brown Produce, Inc. recalls Gala, Fuji, Honeycrisp and Golden Delicious apples due to possible health risk. U.S. Food and Drug Administration, Washington, DC.

[13] U.S. Food and Drug Administration. 2019. North Bay Produce voluntarily recalls fresh apples because of possible health risk. U.S. Food and Drug Administration, Washington, DC.

[14] U.S. Food and Drug Administration. 2017. Fresh Pak Inc. recalls lot specific sliced apple products because of possible health risk. U.S. Food and Drug Administration, Washington, DC.

[15] U.S. Food and Drug Administration. 2020. Country fresh expands voluntary recall. U.S. Food and Drug Administration, Washington, DC.

[16] U.S. Food and Drug Administration. 2017. Draft guidance for industry: control of *Listeria monocytogenes* in ready-to-eat foods. U.S. Food and Drug Administration, Washington, DC.

[17] Tan X, Chung T, Chen Y, Macarisin D, Laborde L, Kovac J. 2019. The occurrence of *Listeria monocytogenes* is associated with built environment microbiota in three tree fruit processing facilities. Microbiome 7:115. doi:10.1186/s40168-019-0726-2. 31431193 PMC6702733

[18] Jorgensen J, Waite Cusic J, Kovacevic J. 2020. Prevalence of *Listeria* spp. in produce handling and processing facilities in the Pacific Northwest. Food Microbiol 90:103468. doi:10.1016/j.fm.2020.103468. 32336359

[19] Estrada EM, Hamilton AM, Sullivan GB, Wiedmann M, Critzer FJ, Strawn LK. 2020. Prevalence, persistence, and diversity of *Listeria monocytogenes* and *Listeria* species in produce packinghouses in three U.S. States. J Food Prot 83:277–286. doi:10.4315/0362-028X.JFP-19-411. 31961227

[20] Sullivan G, Wiedmann M. 2020. Detection and prevalence of *Listeria* in U.S. produce packinghouses and fresh-cut facilities. J Food Prot 83:1656–1666. doi:10.4315/jfp-20-094. 32421820

[21] Alvarez-Ordóñez A, Leong D, Hunt K, Scollard J, Butler F, Jordan K. 2018. Production of safer food by understanding risk factors for *L. monocytogenes* occurrence and persistence in food processing environments. J Food Saf 38:e12516. doi:10.1111/jfs.12516.

[22] Leong D, NicAogáin K, Luque-Sastre L, McManamon O, Hunt K, Alvarez-Ordóñez A, Scollard J, Schmalenberger A, Fanning S, O'Byrne C, Jordan K. 2017. A 3-year multi-food study of the presence and persistence of *Listeria monocytogenes* in 54 small food businesses in Ireland. Int J Food Microbiol 249:18–26. doi:10.1016/j.ijfoodmicro.2017.02.015. 28271853

[23] Murugesan L, Kucerova Z, Knabel SJ, Laborde LF. 2015. Predominance and distribution of a persistent *Listeria monocytogenes* clone in a commercial fresh mushroom processing environment. J Food Prot 78:1988–1998. doi:10.4315/0362-028X.JFP-15-195. 26555522

[24] Cox L, Kleiss T, Cordier J-L, Cordellana C, Konkel P, Pedrazzini C, Beumer R, Siebenga A. 1989. *Listeria* spp. in food processing, non-food and domestic environments. Food Microbiol 6:49–61. doi:10.1016/S0740-0020(89)80037-1.

[25] Strydom A, Vorster R, Gouws PA, Witthuhn RC. 2016. Successful management of *Listeria* spp. in an avocado processing facility. Food Control 62:208–215. doi:10.1016/j.foodcont.2015.10.043.

[26] Leong D, Alvarez-Ordóñez A, Jordan K. 2014. Monitoring occurrence and persistence of *Listeria monocytogenes* in foods and food processing environments in the Republic of Ireland. Front Microbiol 5:436. doi:10.3389/fmicb.2014.00436. 25191314 PMC4138519

[27] Viswanath P, Murugesan L, Knabel SJ, Verghese B, Chikthimmah N, Laborde LF. 2013. Incidence of *Listeria monocytogenes* and *Listeria* spp. in a small-scale mushroom production facility. J Food Prot 76:608–615. doi:10.4315/0362-028X.JFP-12-292. 23575122

[28] Pappelbaum K, Grif K, Heller I, Wüirzner R, Hein I, Ellerbroek L, Wagner M. 2008. Monitoring hygiene on- and at-line is critical for controlling *Listeria monocytogenes* during produce processing. J Food Prot 71:735–741. doi:10.4315/0362-028x-71.4.735. 18468027

[29] Aguado V, Vitas AI, García-Jalón I. 2004. Characterization of *Listeria monocytogenes* and *Listeria innocua* from a vegetable processing plant by RAPD and REA. Int J Food Microbiol 90:341–347. doi:10.1016/s0168-1605(03)00313-1. 14751689

[30] Gianfranceschi M, Gattuso A, Tartaro S, Aureli P. 2003. Incidence of *Listeria monocytogenes* in food and environmental samples in Italy between 1990 and 1999: serotype distribution in food, environmental and clinical samples. Eur J Epidemiol 18:1001–1006. doi:10.1023/a:1025849532417. 14598931

[31] Prazak AM, Murano EA, Mercado I, Acuff GR. 2002. Prevalence of *Listeria monocytogenes* during production and postharvest processing of cabbage. J Food Prot 65:1728–1734. doi:10.4315/0362-028x-65.11.1728. 12430693

[32] Hamilton AM. 2018. Prevalence of indicator organisms, equipment assessment of risk, and lexicon development: an analysis of the tomato packinghouse environment. MS thesis. University of Tennessee, Knoxville, Tennessee.

[33] Lee S, Cetinkaya F, Soyutemiz GE. 2007. Occurrence of *Listeria* species in the processing stages of frozen pepper. J Food Saf 27:134–147. doi:10.1111/j.1745-4565.2007.00067.x.

[34] Macarisin D, Sheth I, Hur M, Wooten A, Kwon HJ, Gao Z, De Jesus A, Jurick W, II, Chen Y. 2019. Survival of outbreak, food, and environmental strains of *Listeria monocytogenes* on whole apples as affected by cultivar and wax coating. Sci Rep 9:12170. doi:10.1038/s41598-019-48597-0. 31434982 PMC6704171

[35] Glass KA, Golden MC, Wanless BJ, Bedale W, Czuprynski C. 2015. Growth of *Listeria monocytogenes* within a caramel-coated apple microenvironment. mBio 6:e01232-15. doi:10.1128/mBio.01232-15. 26463161 PMC4620460

[36] Kenney SJ, Burnett SL, Beuchat LR. 2001. Location of *Escherichia coli* O157:H7 on and in apples as affected by bruising, washing, and rubbing. J Food Prot 64:1328–1333. doi:10.4315/0362-028x-64.9.1328. 11563508

[37] Kenney SJ, Beuchat LR. 2002. Survival of *Escherichia coli* O157:H7 and *Salmonella* Muenchen on apples as affected by application of commercial fruit waxes. Int J Food Microbiol 77:223–231. doi:10.1016/S0168-1605(02)00113-7. 12160082

[38] Ryser ET, Donelly CW. 2015. Listeria, p 425–443. *In* Salfinger Y, Lou Tortorello M (ed), Compendium of methods for the microbiological examination of foods, 5th ed, American Public Health Association, Washington, DC.

[39] Pace International. 2020. Coatings—pome fruit. Pace International, Wapato, WA.

[40] Jo W-S, Song H-Y, Song N-B, Lee J-H, Min S, Bin Song K. 2014. Quality and microbial safety of ‘Fuji’ apples coated with carnauba-shellac wax containing lemongrass oil. LWT - Food Sci Technol 55:490–497. doi:10.1016/j.lwt.2013.10.034.

[41] Lekroengsin S, Keeratipibul S, Trakoonlerswilai K. 2007. Contamination profile of *Listeria* spp. in three types of ready-to-eat chicken meat products. J Food Prot 70:85–89. doi:10.4315/0362-028x-70.1.85. 17265864

[42] Thimothe J, Nightingale KK, Gall K, Scott VN, Wiedmann M. 2004. Tracking of *Listeria monocytogenes* in smoked fish processing plants. J Food Prot 67:328–341. doi:10.4315/0362-028X-67.2.328. 14968966

[43] USDA Food Safety and Inspection Service. 2014. FSIS compliance guideline: controlling *Listeria monocytogenes* in post-lethality exposed ready-to-eat meat and poultry products. USDA Food Safety and Inspection Service, Washington, DC.

[44] Simmons CK, Wiedmann M. 2018. Identification and classification of sampling sites for pathogen environmental monitoring programs for *Listeria monocytogenes*: results from an expert elicitation. Food Microbiol 75:2–17. doi:10.1016/j.fm.2017.07.005. 30056959

[45] Petran RL, Zottola EA. 1989. A study of factors affecting growth and recovery of *Listeria monocytogene*s Scott A. J Food Sci 54:458–460. doi:10.1111/j.1365-2621.1989.tb03105.x.

[46] Pietrysiak E, Kummer J, Hanrahan I, Ganjyal G. 2020. Hurdle effect of hot air impingement drying and surfactant-sanitizer wash on removal of *Listeria innocua* from fresh apples. J Food Prot 83:1488–1494. doi:10.4315/JFP-20-078. 32311702

[47] Chaturongkasumrit Y, Takahashi H, Keeratipibul S, Kuda T, Kimura B. 2011. The effect of polyesterurethane belt surface roughness on *Listeria monocytogenes* biofilm formation and its cleaning efficiency. Food Control 22:1893–1899. doi:10.1016/j.foodcont.2011.04.032.

[48] Gazula H, Quansah J, Allen R, Scherm H, Li C, Takeda F, Chen J. 2019. Microbial loads on selected fresh blueberry packing lines. Food Control 100:315–320. doi:10.1016/j.foodcont.2019.01.032.

[49] Meireles A, Fulgêncio R, Machado I, Mergulhão F, Melo L, Simões M. 2017. Characterization of the heterotrophic bacteria from a minimally processed vegetables plant. LWT - Food Sci Technol 85:293–300. doi:10.1016/j.lwt.2017.01.038.

[50] Blatter S, Giezendanner N, Stephan R, Zweifel C. 2010. Phenotypic and molecular typing of *Listeria monocytogenes* isolated from the processing environment and products of a sandwich-producing plant. Food Control 21:1519–1523. doi:10.1016/j.foodcont.2010.04.025.

[51] U.S. Food and Drug Administration. 2015. External summary report: FY 2013 inspection, environmental sampling and sample collection (pre and post-process) at cantaloupe packinghouses assignment (DFPG #13–19). U.S. Food and Drug Administration, Washington, DC.

[52] Blackman IC, Frank JF. 1996. Growth of *Listeria monocytogenes* as a biofilm on various food-processing surfaces. J Food Prot 59:827–831. doi:10.4315/0362-028X-59.8.827. 31159129

[53] Zoellner C, Aguayo-Acosta A, Wasim Siddiqui M, Davila-Avina J. 2018. Peracetic acid in disinfection of fruits and vegetables, p 53–66. *In* Postharvest disinfection of fruits and vegetables. Elsevier, Philadelphia, PA.

[54] Pietrysiak E, Smith S, Ganjyal G. 2019. Food safety interventions to control *Listeria monocytogenes* in the fresh apple packing industry: a review. Compr Rev Food Sci Food Saf 18:1705–1726. doi:10.1111/1541-4337.12496. 33336959

[55] Brackett RE. 1987. Antimicrobial effect of chlorine on *Listeria monocytogenes*. J Food Prot 50:999–1003. doi:10.4315/0362-028X-50.12.999. 30978827

[56] Webb C, Erickson M, Davey L, Doyle M. 2015. Evaluation of single or double hurdle sanitizer applications in simulated field or packing shed operations for cantaloupes contaminated with *Listeria monocytogenes*. Agriculture 5:231–244. doi:10.3390/agriculture5020231.

[57] Zhou B, Luo Y, Nou X, Lyu S, Wang Q. 2015. Inactivation dynamics of *Salmonella enterica*, *Listeria monocytogenes*, and *Escherichia coli* O157:H7 in wash water during simulated chlorine depletion and replenishment processes. Food Microbiol 50:88–96. doi:10.1016/j.fm.2015.03.004. 25998820

[58] Williams K. 2018. Fate of Listeria monocytogenes on intact and wounded apples during storage and in a simulated dump tank. MS thesis. The Pennsylvania State University, State College, PA.

[59] Baert L, Vandekinderen I, Devlieghere F, Van Coillie E, Debevere J, Uyttendaele M. 2009. Efficacy of sodium hypochlorite and peroxyacetic acid to reduce murine norovirus 1, B40-8, *Listeria monocytogenes*, and *Escherichia coli* O157:H7 on shredded iceberg lettuce and in residual wash water. J Food Prot 72:1047–1054. doi:10.4315/0362-028x-72.5.1047. 19517733

[60] Banach JL, Sampers I, Van Haute S, van der Fels-Klerx HJ. 2015. Effect of disinfectants on preventing the cross-contamination of pathogens in fresh produce washing water. Int J Environ Res Public Health 12:8658–8677. doi:10.3390/ijerph120808658. 26213953 PMC4555240

[61] Rodgers SL, Cash JN, Siddiq M, Ryser ET. 2004. A comparison of different chemical sanitizers for inactivating *Escherichia coli* O157:H7 and *Listeria monocytogenes* in solution and on apples, lettuce, strawberries, and cantaloupe. J Food Prot 67:721–731. doi:10.4315/0362-028x-67.4.721. 15083724

[62] Krysinski EP, Brown LJ, Marchisello TJ. 1992. Effect of cleaners and sanitizers on *Listeria monocytogenes* attached to product contact surfaces. J Food Prot 55:246–251. doi:10.4315/0362-028X-55.4.246. 31071783

[63] Shen X, Su Y, Hua Z, Cong J, Dhowlaghar N, Sun Q, Lin S, Green T, Perrault M, Galeni M, Hanrahan I, Suslow TV, Zhu M-J. 2020. Verification of peroxyacetic acid treatment against *L. monocytogenes* on fresh apples using *E. faecium* NRRL B-2354 as a surrogate in commercial spray-bar operations. Food Microbiol 92:103590. doi:10.1016/j.fm.2020.103590. 32950134

[64] Beuchat LR, Nail BV, Adler BB, Clavero MRS. 1998. Efficacy of spray application of chlorinated water in killing pathogenic bacteria on raw apples, tomatoes, and lettuce. J Food Prot 61:1305–1311. doi:10.4315/0362-028x-61.10.1305. 9798146

[65] United Fresh Produce Association. 2018. Strategies for *Listeria* control in tree fruit packinghouses. United Fresh Produce Association, Washington, DC.

[66] Smith A, Moorhouse E, Monaghan J, Taylor C, Singleton I. 2018. Sources and survival of *Listeria monocytogenes* on fresh, leafy produce. J Appl Microbiol 125:930–942. doi:10.1111/jam.14025. 30039586

[67] Spotts RA, Cervantes LA, Mielke EA. 1999. Variability in postharvest decay among apple cultivars. Plant Dis 83:1051–1054. doi:10.1094/PDIS.1999.83.11.1051. 30841275

[68] Conway WS, Leverentz B, Saftner RA, Janisiewicz WJ, Sams CE, Leblanc E. 2000. Survival and growth of *Listeria monocytogenes* on fresh-cut apple slices and its interaction with *Glomerella cingulata* and *Penicillium expansum*. Plant Dis 84:177–181. doi:10.1094/PDIS.2000.84.2.177. 30841311

[69] Burnett SL, Chen J, Beuchat LR. 2000. Attachment of *Escherichia coli* O157:H7 to the surfaces and internal structures of apples as detected by confocal scanning laser microscopy. Appl Environ Microbiol 66:4679–4687. doi:10.1128/aem.66.11.4679-4687.2000. 11055910 PMC92366

[70] Harris LJ, Farber JN, Beuchat LR, Parish ME, Suslow TV, Garrett EH, Busta FF. 2003. Outbreaks associated with fresh produce: incidence, growth, and survival of pathogens in fresh and fresh-cut produce. Comp Rev Food Sci Food Safety 2:78–141. doi:10.1111/j.1541-4337.2003.tb00031.x.

[71] Walker S, Archer P, Banks J. 1990. Growth of *Listeria monocytogenes* at refrigeration temperatures. J Appl Bacteriol 68:157–162. doi:10.1111/j.1365-2672.1990.tb02561.x. 2108109

[72] Becker LA, Evans SN, Hutkins RW, Benson AK. 2000. Role of ς*B* in adaptation of *Listeria monocytogenes* to growth at low temperature. J Bacteriol 182:7083–7087. doi:10.1128/JB.182.24.7083-7087.2000. 11092874 PMC94839

[73] Gandhi M, Chikindas ML. 2007. *Listeria*: a foodborne pathogen that knows how to survive. Int J Food Microbiol 113:1–15. doi:10.1016/j.ijfoodmicro.2006.07.008. 17010463

[74] Beales N. 2004. Adaptation of microorganisms to cold temperatures, weak acid preservatives, low pH, and osmotic stress: a review. Compr Rev Food Sci Food Saf 3:1–20. doi:10.1111/j.1541-4337.2004.tb00057.x. 33430556

[75] Sheng L, Edwards K, Tsai H, Hanrahan I, Zhu M. 2017. Fate of *Listeria monocytogenes* on fresh apples under different storage temperatures. Front Microbiol 8:1396. doi:10.3389/fmicb.2017.01396. 28790993 PMC5522875

[76] Sheng L, Hanrahan I, Sun X, Taylor MH, Mendoza M, Zhu MJ. 2018. Survival of *Listeria innocua* on Fuji apples under commercial cold storage with or without low dose continuous ozone gaseous. Food Microbiol 76:21–28. doi:10.1016/j.fm.2018.04.006. 30166144

[77] Juhneviča K, Skudra G, Skudra L. 2011. Evaluation of microbiological contamination of apple fruit stored in a modified atmosphere. Environ Exp Biol 9:53–59.

[78] Ruiz-Llacsahuanga B, Hamilton A, Zaches R, Hanrahan I, Critzer F. 2021. Utility of rapid tests to assess the prevalence of indicator organisms (aerobic plate count, *Enterobacteriaceae*, coliforms, *Escherichia coli*, and *Listeria* spp.) in apple packinghouses. Int J Food Microbiol 337:108949. doi:10.1016/j.ijfoodmicro.2020.108949. 33220648

[79] Hitchins A, Jinneman K, Chen Y. 2017. Detection of *Listeria monocytogenes* in foods and environmental samples, and enumeration of *Listeria monocytogenes* in foods. *In* Bacteriological analytical manual. U.S. Food and Drug Administration, Washington, DC.

