# Supplementary material for: Prevalence of Listeria Species on Food Contact Surfaces in Washington State Apple Packinghouses
Source: Appl Environ Microbiol. 2021 Apr 13;87(9):e02932-20. doi: 10.1128/AEM.02932-20 (PMC8091025; doi:10.1128/AEM.02932-20)

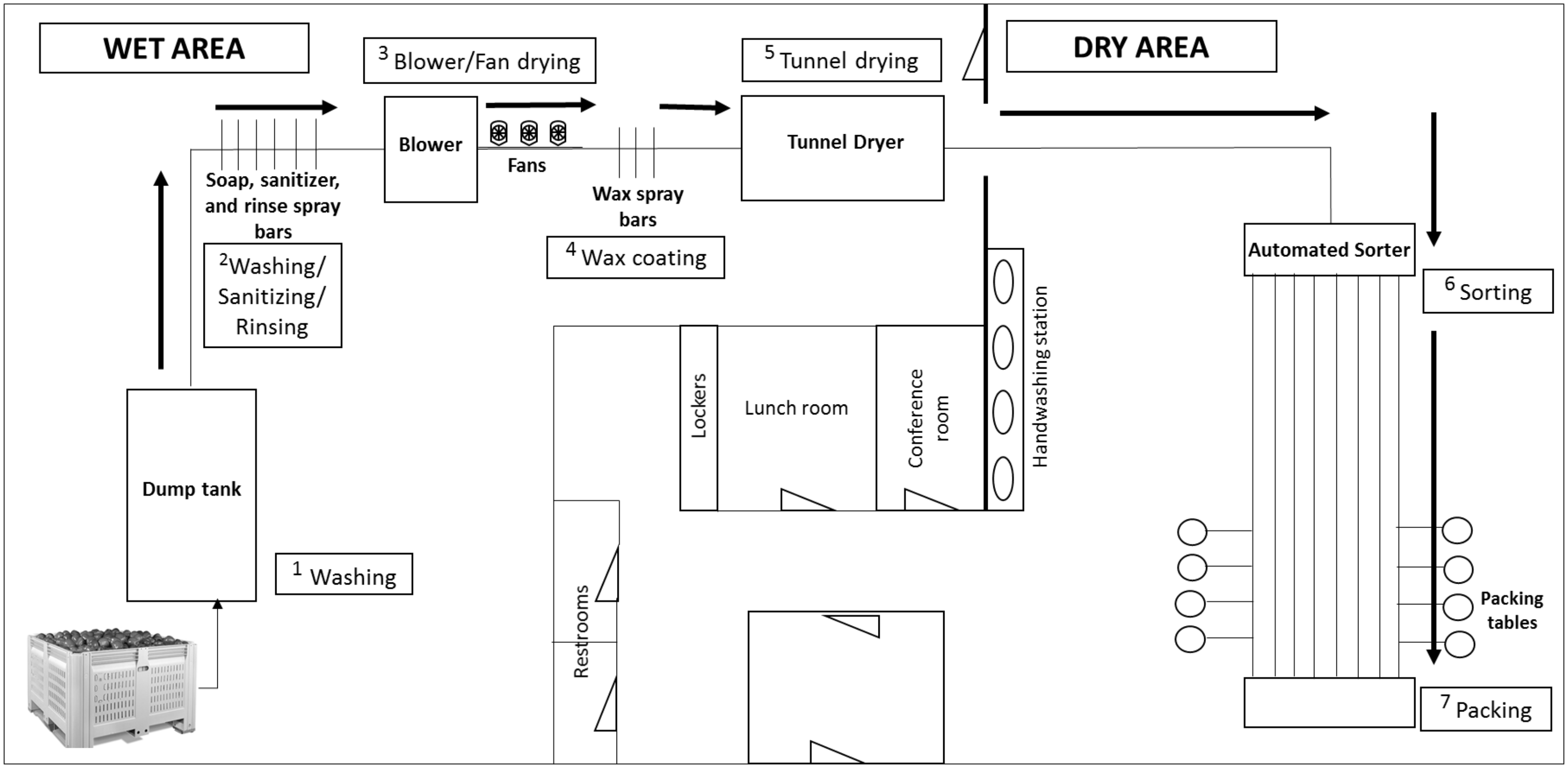

Supplemental Figure 1. Example apple packinghouse flow diagram.

Supplemental Table 1. Examples of food contact surfaces tested in apple packinghouses

1. Brush rollers	2. Polishing brushes
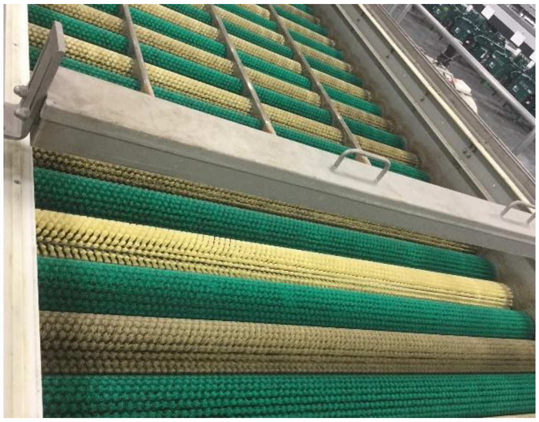	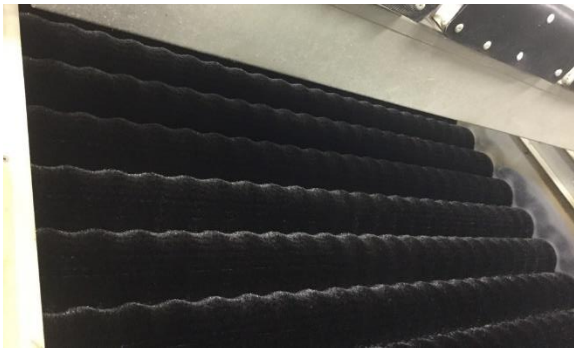
3. Bristle rollers	4. Polyvinylchloride (PVC) rollers
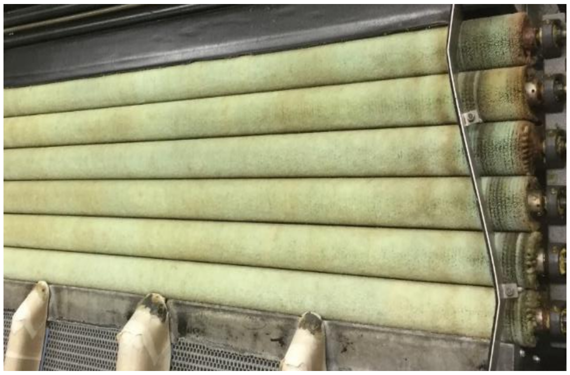	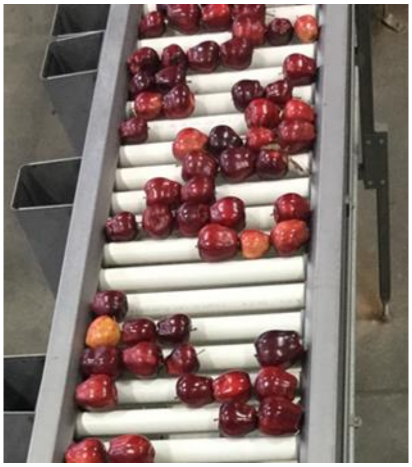
5. Dryer rollers	6. Sorting brushes
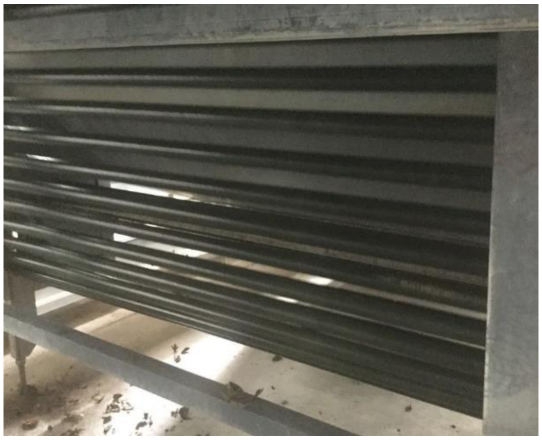	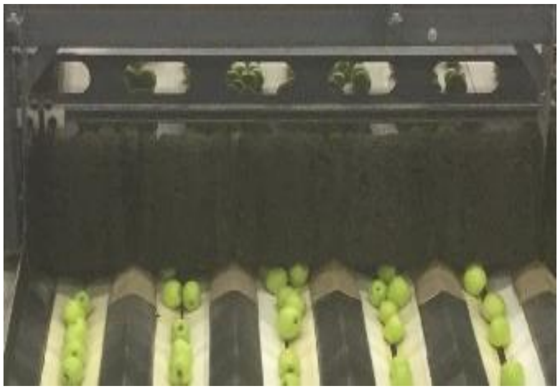

7. Interlocking conveyor belts	8. Solid conveyor belts
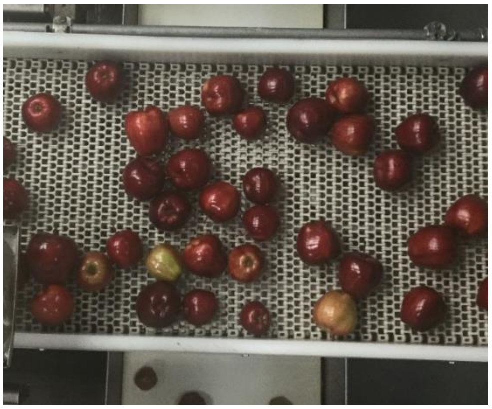	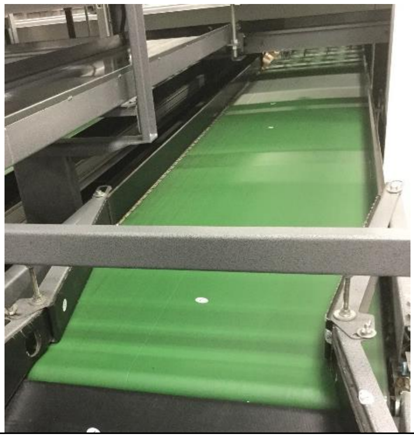
9. Plastic guide rails	10. Sorter cups and cup droppers
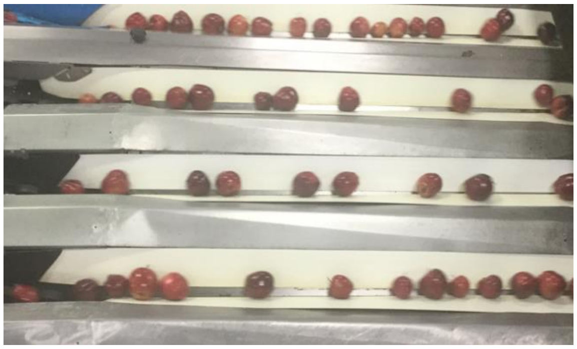	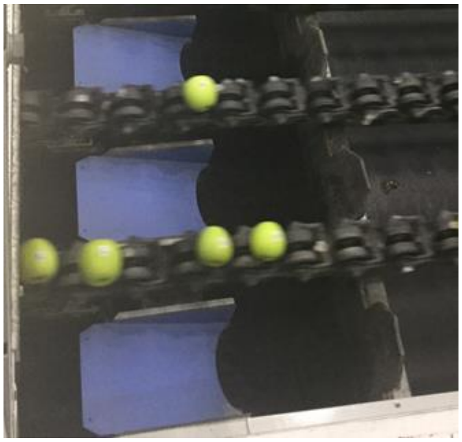
11. Dump tank and flumes	12. Dividers
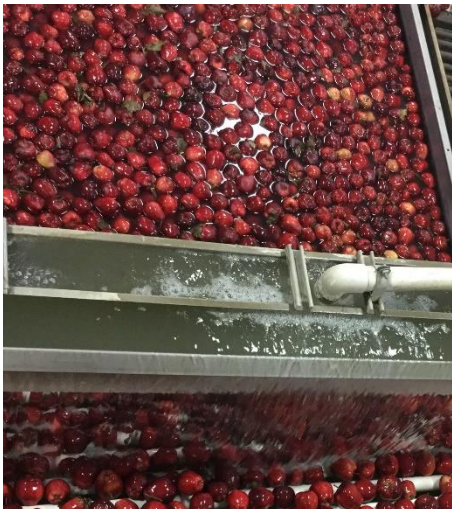	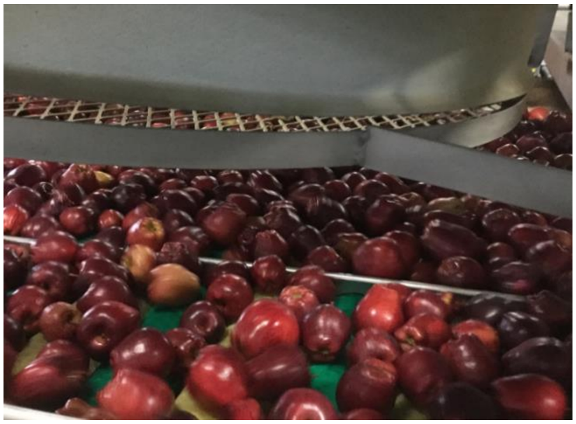

13. Teflon tapes and transfer points

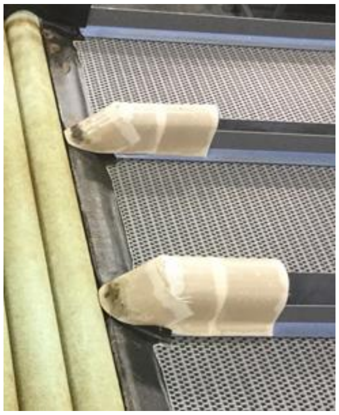
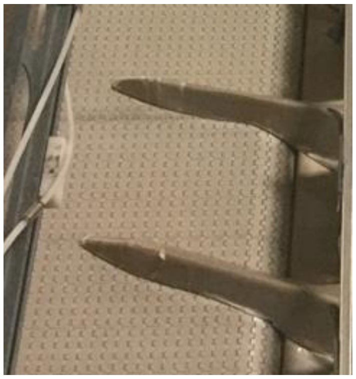
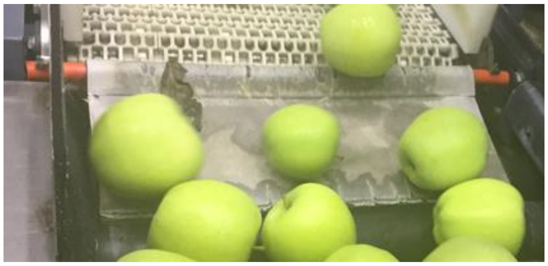
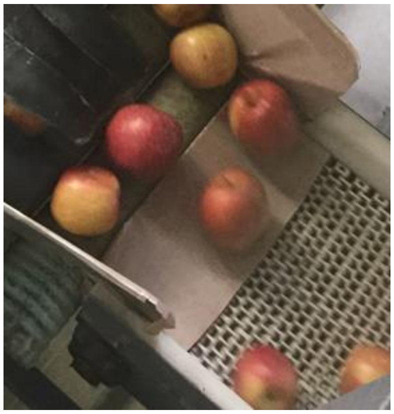

14. Plastic flaps and transfer points

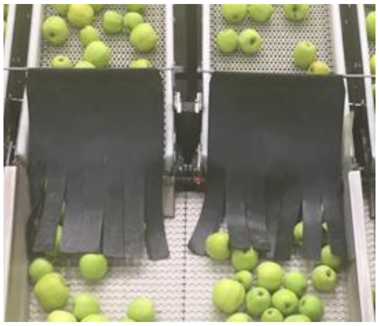
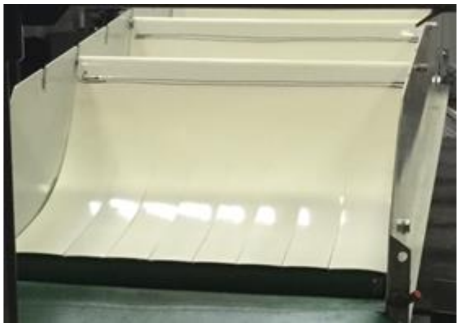
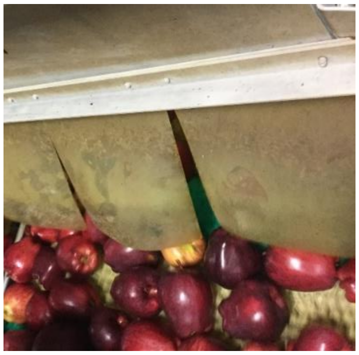
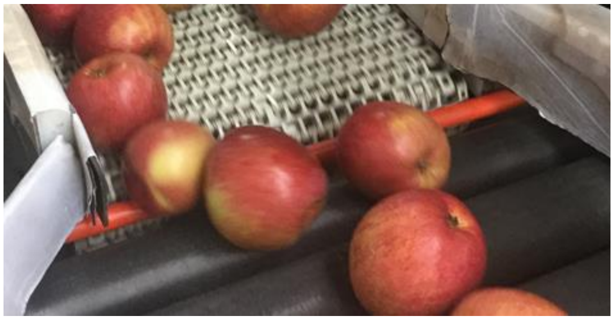
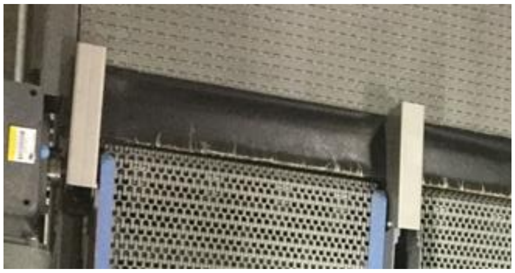

Supplement: Supplemental file 1 [file AEM.02932-20-s0001.pdf]
